# Identification of diagnostic biomarkers for osteoarthritis through bioinformatics and machine learning

**DOI:** 10.1016/j.heliyon.2024.e27506

**Published:** 2024-03-07

**Authors:** KunPeng Wang, Ye Li, JinXiu Lin

**Affiliations:** Department of Orthopedics, Zibo First Hospital, Zibo, 255200, China

**Keywords:** Osteoarthritis, Differentially expressed genes, Enrichment analysis, Co-expression modules, Characteristic genes, Immune cell infiltration

## Abstract

Osteoarthritis (OA) is a prevalent degenerative joint disease characterized by cartilage degradation, inflammatory arthritis, and joint dysfunction. Currently, there is a lack of effective early diagnostic methods and treatment strategies for OA. Bioinformatics and biomarker research provide new possibilities for early detection and personalized therapy of OA. In this study, we investigated the molecular mechanisms of OA and important signaling pathways involved in disease progression through bioinformatics analysis. Firstly, using the limma package, we analyzed the differentially expressed genes (DEGs) between normal healthy samples and OA cartilage tissue samples. These DEGs were found to be primarily involved in biological processes such as extracellular matrix (ECM) binding, immune receptor activity, and cytokine activity, as well as signaling pathways including cytokine receptors, ECM-receptor interaction, and PI3K-Akt. Gene set enrichment analysis revealed that in the OA group, signaling pathways such as AMPK, B cell receptor, IL-17, and PPAR were downregulated, while calcium signaling pathway, cell adhesion molecules, ECM-receptor interaction, TGF-β signaling pathway, and Wnt signaling pathway were upregulated. Additionally, we constructed a co-expression module network using WGCNA and identified key modules associated with OA, from which we selected 7 most predictive OA characteristic genes. Among them, ANTXR1, KCNS3, SGCD, and LIN7A were correlated with the level of immune cell infiltration. This study elucidates the mechanisms underlying the development of OA and identifies potential diagnostic markers and therapeutic targets, providing important insights for early diagnosis and treatment of OA.

## 1 Introduction

Osteoarthritis (OA) is a prevalent chronic degenerative joint disease primarily affecting the articular cartilage and adjacent tissues. This disease poses a major health challenge globally, particularly among the elderly, significantly impairing patients' quality of life and generating considerable economic costs [1]. The main features of OA include cartilage degradation, osteophyte formation, synovial inflammation, subchondral bone thickening, and joint space narrowing. A multitude of factors including age, gender, obesity, traumatic injuries, stress-induced damage, and congenital joint abnormalities contribute to the development of OA [[2], [3], [4]]. Currently, the treatment of OA primarily involves nonsteroidal anti-inflammatory drugs (NSAIDs) and joint replacement surgeries. Therefore, early detection, diagnosis, and timely intervention are crucial to enhance OA prognosis [5,6].

The diagnosis of OA is predominantly based on clinical symptoms, physical signs, and imaging examinations [7]. Recognizing OA in its incipient stages allows for timely intervention and management, thereby altering the disease trajectory [8]. However, these methods are limited by low sensitivity in early-stage lesions detection and an inability to precisely predict disease progression and therapeutic response. Consequently, identifying reliable biomarkers for supporting the diagnosis and assessment of OA progression is critically important. Research has indicated an association between specific serum molecules and the occurrence and severity of OA [9]. For instance, CS846, a proteoglycan component, exhibits elevated levels in patients with OA [10]. Additionally, the inflammatory marker called C-reactive protein (CRP) is associated with the development and severity of OA-related arthritis [11,12]. Measurement of these serum biomarkers aids physicians in evaluating the condition of OA patients, monitoring disease progression, and assessing treatment efficacy. Besides serum biomarkers, molecules in synovial fluid are also pertinent to OA. Higher levels of C-telopeptide of type II collagen (CTX-II), a collagen degradation product, have been observed in early-stage OA patients, indicating its potential as an OA marker [13]. Moreover, the presence of cartilage oligomeric matrix protein (COMP) in synovial fluid is associated with the occurrence and severity of OA [[14], [15], [16]]. Apart from serum and synovial fluid markers, urine metabolites have demonstrated potential in predicting and monitoring disease progression in OA. A metabolomics analysis of urine samples from OA patients identified metabolites, including arginine, correlated with the severity and progression of OA [17]. This indicates the potential of urine metabolites as non-invasive biomarkers for OA.

Recently, bioinformatics and machine learning have emerged as powerful tools widely employed in biomedical research. Bioinformatics and machine learning synergistically contribute to the advancement of biomedical research by providing powerful tools for data analysis, predictive modeling, and the discovery of meaningful patterns in complex biological datasets. The integration of these technologies holds great promise in unlocking new insights into diseases and improving patient outcomes [18,19]. This study aimed to utilize bioinformatics and machine learning techniques to identify diagnostic biomarkers associated with OA and explore their potential applications in early diagnosis and personalized treatment. Publicly available gene expression profile datasets were analyzed to identify differentially expressed genes (DEGs) between OA and normal tissues. Functional annotation tools were employed to elucidate the biological functions of these genes. Furthermore, weighted gene co-expression network analysis (WGCNA) was used to identify key modules most strongly correlated with OA, from which crucial targets were selected. Subsequently, predictive models, utilizing the support vector machine recursive feature elimination (SVM-RFE) algorithm and the least absolute shrinkage and selection operator (LASSO) regression algorithm, were constructed to identify potential diagnostic biomarkers for OA. The accuracy of the models was evaluated using receiver operating characteristic (ROC) curves, and the correlation between biomarkers and clinical features was explored. By identifying reliable biomarkers, we can enhance the predictive accuracy of OA and provide a basis for physicians to develop more effective treatment strategies. Furthermore, deeper insight into OA pathogenesis and its associated pathways aids in identifying new therapeutic targets and advancing drug development, thereby bolstering precision medicine.

## 2 Materials and methods

### 2.1 Data collection and preprocessing

We searched the Gene Expression Omnibus (GEO) database (https://www.ncbi.nlm.nih.gov/geo/) using the keyword “osteoarthritis” to retrieve OA expression profile datasets. The samples for OA mainly originated from three tissues: cartilage (GSE51588 and GSE114007), synovium (GSE12021_GPL96, GSE55457, GSE82107), and peripheral blood (GSE63359). The details of the datasets are summarized in Table 1. Standard data processing procedures were applied to the GEO datasets, which involved converting probes to gene symbols, removing redundant probes targeting the same gene, and averaging multiple probes mapped to the same symbol. To minimize batch effects, we performed batch correction using the SVA package for the synovial samples (GSE12021_GPL96, GSE55457, and GSE82107). The batch-corrected synovial data were merged into a single large dataset, which was combined with GSE114007 and GSE63359 as the validation set. GSE51588 dataset served as the training set. Differential expression analysis between normal and OA samples in the GSE51588 dataset was conducted using the limma package. DEGs were filtered based on a threshold of false discovery rate (FDR) < 0.05 and |log2FC|>1, resulting in the final set of DEGs.

**Table 1:** Summary of the analyzed datasets.

ID	Control	OA	Tissue
GSE51588	10	40	Cartilage
GSE114007	18	20	Cartilage
GSE12021_GPL96	9	10	Synovium
GSE55457	10	10	Synovium
GSE82107	7	10	Synovium
GSE63359	26	46	Blood

### 2.2 Pathway and biological function enrichment analysis

KEGG analysis was performed to identify signaling pathways associated with the DEGs in OA. Additionally, GO functional enrichment analysis was conducted to explore the biological functions of the identified DEGs, including molecular function (MF), cellular component (CC), and biological process (BP). To study activated signaling pathways and biological functions in OA, gene set enrichment analysis (GSEA) was performed. A p-value <0.05 was considered statistically significant.

### 2.3 Construction of Co-expression networks

WGCNA, a systematic biological approach, is utilized to describe gene correlation patterns among different samples. It can be used to identify highly coordinated sets of genes and potential biomarkers based on the interconnections between gene sets and phenotypes. We conducted WGCNA analysis using the top 25% genes ranked by coefficient of variation from the GSE51588 expression profile. Firstly, a sample clustering tree was generated to assess the presence of outliers. Then, the adjacency matrix (AM) was transformed into a Topological Overlap Measure (TOM) matrix (scale-free R^2 = 0.85). Next, the “DynamicTreeCut” method was applied to assign genes with similar expression profiles to the same gene modules (with parameter adjustment minModuleSize = 50). Finally, the correlation between different modules and the pathogenesis of OA was calculated, with the most correlated module being selected as the candidate genes derived from WGCNA.

### 2.4 Selection of feature genes

LASSO regression analysis allows for variable selection and complexity regularization in prediction variables to achieve accurate predictions. To identify genes of significant importance in OA diagnosis, we performed LASSO logistic regression analysis using the glmnet package on the intersection genes between DEGs and WGCNA modules.

Relationship between Feature Genes and Clinical Characteristics, and Construction of Column Line Plots.

Patients were categorized into different subgroups based on distinct clinical and pathological features, including age (>65 and≤65) and gender (female and male). The expression of feature genes was compared among different clinical groups using the wilcoxn.test. We constructed column line plot models using the rms package to predict the risk of OA. Subsequently, we validated the diagnostic performance of candidate biomarkers by constructing ROC curves using the pROC package. The area under the ROC curve (AUC) was used as a measure of accuracy. We employed a standard to differentiate excellent accuracy (0.9 ≤ AUC <1), good accuracy (0.8 ≤ AUC <0.9), and no information accuracy (AUC = 0.5).

### 2.5 Validation of diagnostic value of feature genes

To evaluate the diagnostic accuracy of these feature genes for OA, ROC curves were generated using data from an independent validation cohort. The AUC accurately reflects the effectiveness of feature genes as diagnostic biomarkers. Differential expression of the selected diagnostic genes was also validated in three independent validation cohorts.

### 2.6 Relationship between feature genes and immune cell infiltration

The CIBERSORT algorithm (http://cibersort.stanford.edu/) is a gene expression-based deconvolution method used to assess the variation of a specific gene set relative to the rest of the genes in a sample. Leveraging the CIBERSORT algorithm, we analyzed the differences in immune cell infiltration between OA and normal cartilage tissues, consisting of 22 immune cell subsets. We evaluated the association between these immune cells and the expression of feature genes in OA samples. A |cor| > 0.5 and p-value <0.05 were considered indicative of correlation.

### 2.7 Statistical analysis

All analyses were conducted using R version 4.1.2. When assessing the significance between various values such as gene expression, infiltration proportions, and other features, the Wilcoxon rank-sum test was employed to compare differences between two groups of samples. For visual representation, “ns” represents p > 0.05, "*" represents p < 0.05, "**" represents p < 0.01, "***" represents p < 0.001, and "****" represents p < 0.0001.

## 3 Results

### 3.1 Identification of DEGs in OA

**Fig. 1:**
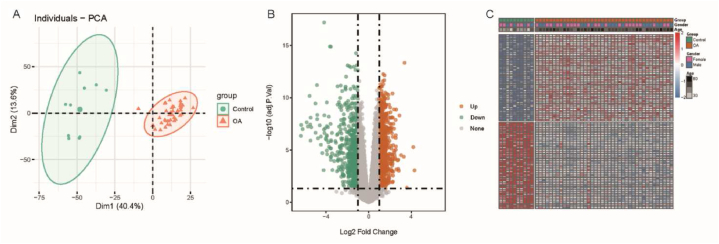
Identification of DEGs in OA; (A) PCA analysis of normal control and articular cartilage samples from the GSE51588 dataset; (B) Volcano plot showing the DEGs; (C) Heatmap displaying the DEGs.

We used the limma package to analyze the DEGs between normal samples and OA cartilage tissue samples. After data filtering, we identified 1591 DEGs, including 950 upregulated genes and 641 downregulated genes. To begin with, we performed principal component analysis (PCA) on the normal and OA cartilage tissue samples, which revealed distinct clustering of each group, indicating significant differences between the two groups of samples (Fig. 1A). The volcano plot displayed the distribution of upregulated and downregulated DEGs (Fig. 1B). Furthermore, we presented a heat map depicting the top 50 upregulated and downregulated genes (Fig. 1C), further highlighting their differential expression patterns among the samples.

### 3.2 Enrichment analysis of DEGs

**Fig. 2:**
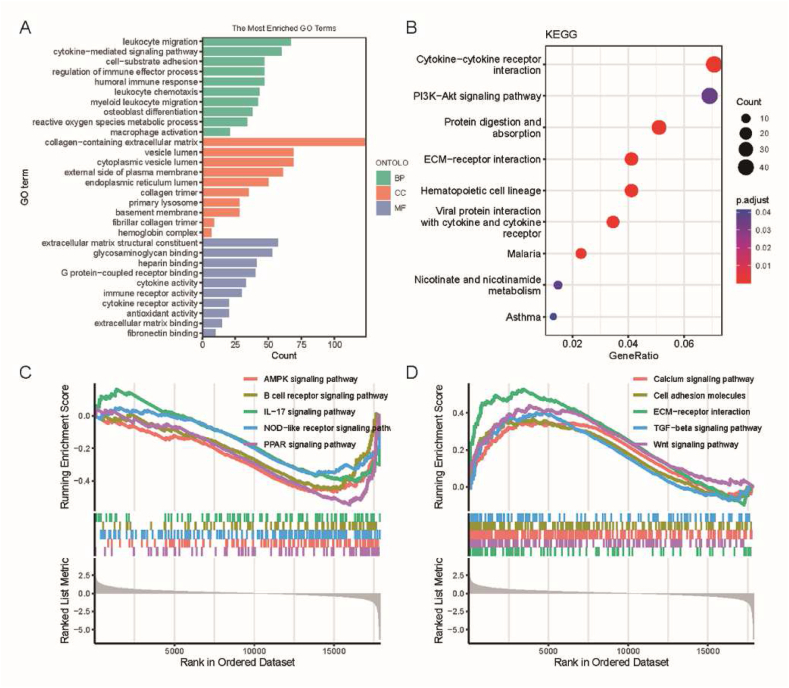
Gene set enrichment analysis; (A) GO enrichment analysis of DEGs; (B) KEGG pathway enrichment analysis of DEGs; (C) Suppressed signaling pathways in the OA group; (D) Activated signaling pathways in the OA group.

We performed gene ontology (GO) and Kyoto Encyclopedia of Genes and Genomes (KEGG) enrichment analyses on the DEGs to gain insights into the signaling pathways and biological functions in which these genes are involved. The results of GO enrichment analysis revealed that the DEGs were primarily enriched in the basement membrane, lysosome, and endoplasmic reticulum lumen, indicating their involvement in biological processes such as ECM binding, immune receptor activity, and cytokine activity, contributing to humoral immune response, osteoblast differentiation, leukocyte migration, and reactive oxygen species metabolism (Fig. 2A). KEGG enrichment analysis demonstrated that the DEGs were predominantly enriched in cytokine receptors, ECM-receptor interaction, and the PI3K-Akt signaling pathway (Fig. 2B). Gene set enrichment analysis (GSEA) allows for a comprehensive and systematic exploration of differences in biological behaviors and functional pathways between different groups using complete sets of gene expression data. Through GSEA analysis of the entire gene expression dataset, we observed significant inhibition of AMPK, B cell receptor, IL-17, and PPAR signaling pathways in the OA group compared to the normal group, while calcium signaling pathway, cell adhesion molecules, ECM-receptor interaction, TGF-beta signaling pathway, and Wnt signaling pathway were significantly activated (Fig. 2C and D). Enrichment analysis provides a more comprehensive and systematic understanding of the biological behavior of DEGs in OA from a functional and pathway perspective. These findings suggest that the regulation of the aforementioned signaling pathways plays a crucial role in OA progression, cellular activities, and inflammatory responses.

### 3.3 Construction of WGCNA network to identify potential OA-related gene modules

**Fig. 3:**
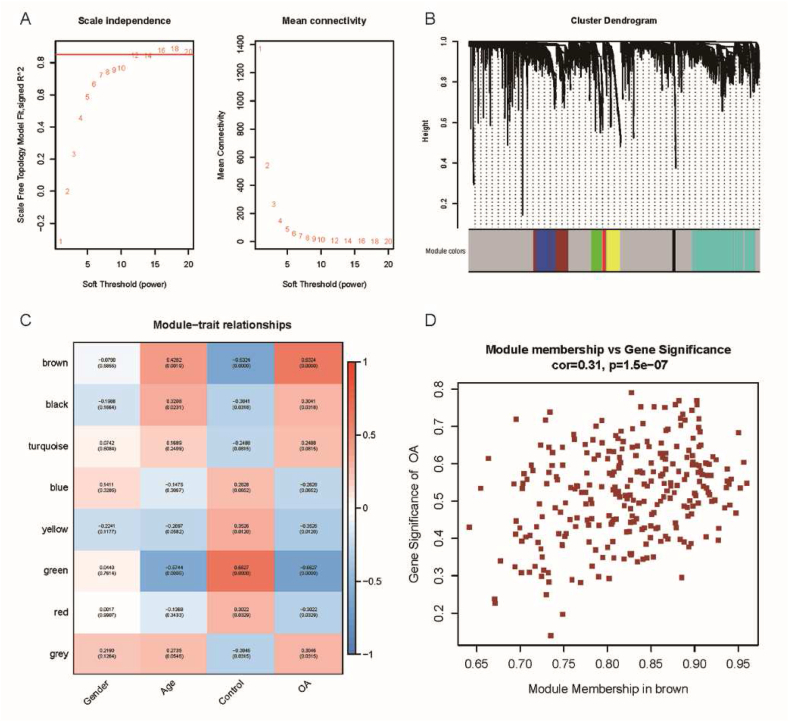
Construction of WGCNA network; (A) Scale-free fitting index and mean connectivity for different soft thresholding values; (B) Gene clustering dendrogram, where genes with similar expression patterns are grouped together and represented by the same color; (C) Correlation between different modules and OA; (D) Scatter plot showing the feature genes in the orange-red module. (For interpretation of the references to color in this figure legend, the reader is referred to the Web version of this article.)

In order to identify potential gene modules associated with OA, WGCNA was performed. Employing the WGCNA algorithm, a co-expression module network was constructed, and modules associated with OA were identified. During the analysis, we set the soft threshold to 16 to ensure strong overall connectivity within the co-expression modules (Fig. 3A). Ultimately, we established eight co-expression modules represented by different colors, clustering genes with similar expression patterns within each module, revealing the gene correlation structure within the modules (Fig. 3B). The orange-red module, shown in Fig. 3C, exhibited a positive correlation with OA. Therefore, we identified the orange-red module as a key module for further analysis and selected 275 genes within it (Fig. 3C and D). The results of WGCNA analysis provide initial insights into OA-related gene modules, suggesting that genes within the key module may play a significant role in the pathogenesis and progression of OA.

### 3.4 Selection of OA-related feature genes

**Fig. 4:**
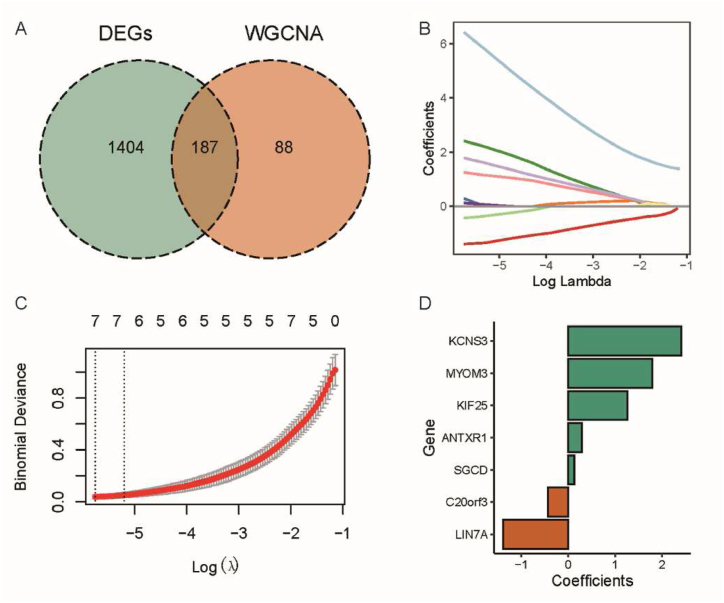
Selection of OA feature genes; Intersection of DEGs and genes from WGCNA; (B) Trajectories of the seven feature genes, with the x-axis representing the log value of the independent variable lambda, and different colors indicating different genes; (C) Confidence intervals at different lambdas; (D) Correlation coefficients for each gene. (For interpretation of the references to color in this figure legend, the reader is referred to the Web version of this article.)

An intersection analysis between the DEGs and key genes within the WGCNA orange-red module was performed using a Venn diagram to further screen for candidate genes associated with OA. We identified 187 overlapping genes as potential candidate genes that may play important roles in the development and progression of OA (Fig. 4A). Through LASSO logistic regression analysis, we determined seven genes with the highest predictive power among these candidates. These genes are LIN7A, KIF25, MYOM3, ANTXR1, KCNS3, SGCD, and C20orf3 (Fig. 4B and C). The correlation coefficients represent the linear relationship between the feature genes and OA. Higher correlation coefficients indicate a strong association between the feature genes and OA, while lower correlation coefficients suggest a weaker association. The results indicate that KCNS3 exhibits the highest correlation with OA (Fig. 4D). These findings provide important information for further investigating the association and potential roles of the feature genes in OA.

### 3.5 Diagnostic value of OA feature genes

**Fig. 5:**
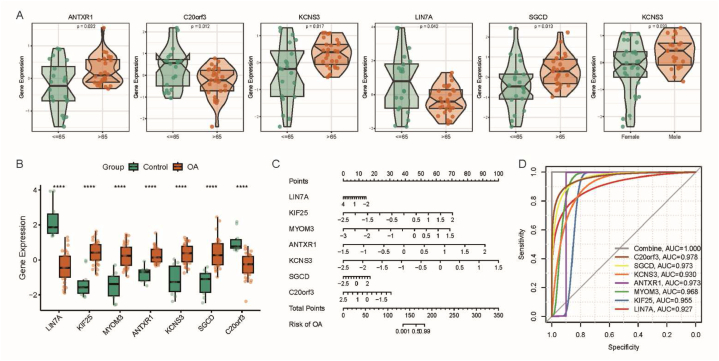
Diagnostic value of OA feature genes; (A) Expression of feature genes in different clinical subgroups; (B) Differential expression of feature genes between normal control and OA cartilage samples; (C) Column line plot model of feature genes; (D) ROC curve evaluating the diagnostic performance of the column line plot model and each feature gene.

We further analyzed the expression patterns of the OA feature genes in different clinical groups. The results showed significant differences in gene expression among different age groups, except for KIF25 and MYOM3. Among these genes, ANTXR1, KCNS3, SGCD, and KCNS3 exhibited significantly higher expression in female samples compared to male samples (Fig. 5A). Furthermore, compared to the normal group, KIF25, MYOM3, ANTXR1, KCNS3, and SGCD showed higher expression levels in OA (Fig. 5B). Subsequently, we constructed a column line plot model to predict the risk of OA (Fig. 5C). This model accurately predicts the risk based on clinical data and the expression levels of the feature genes. We calculated the ROC curves for the seven feature genes and the column line plot model to evaluate their diagnostic performance (Fig. 5D). According to the established criteria, an area under the ROC curve (AUC) of 0.9≤AUC<1 indicates excellent accuracy, 0.8≤AUC<0.9 indicates good accuracy, while AUC = 0.5 indicates no informative accuracy. Based on these criteria, our column line plot model effectively distinguishes OA from the control group, and its diagnostic value surpasses that of individual feature genes. To validate the diagnostic efficacy of the feature genes, we used data from GSE114007 (cartilage tissue), GSE63359 (blood), and batch-corrected synovial tissue. The results demonstrated high expression of MYOM3, ANTXR1, KCNS3, and SGCD in cartilage tissue (Supplementary Fig. 1A), high expression of KIF25, ANTXR1, KCNS3, and SGCD in blood (Supplementary Fig. 1B), and high expression of KIF25, ANTXR1, KCNS3, and SGCD in synovial tissue (Supplementary Fig. 1C). These experimental findings suggest the crucial involvement of ANTXR1, KCNS3, and SGCD in OA.

### 3.6 Association between OA feature genes and immune cell infiltration

Based on the results of GO enrichment and KEGG pathway analysis, DEGs were primarily associated with inflammatory responses. Therefore, we further investigated the levels of immune cell infiltration in normal control and OA joint cartilage tissue using the CIBERSORT algorithm. By applying the CIBERSORT algorithm, we revealed the proportions of 22 immune cell types in normal control and OA tissues, visualizing the results through a percentage stacked bar plot (Supplementary Fig. 2A). The results demonstrated that in the OA group, the proportions of Plasma cells, T cells CD4 memory resting, T cells CD4 memory activated, NK cells resting, Monocytes, Eosinophils, and Neutrophils were significantly lower compared to the normal control group. On the other hand, the proportions of T cells CD4 naive, NK cells activated, and other cells were significantly higher in the OA group (Supplementary Fig. 2B). Further correlation analysis revealed a significant negative correlation (correlation coefficient <0.5) between the expression of ANTXR1, KCNS3, SGCD genes, and T cells CD8, suggesting potential roles for these genes in inhibiting or regulating T cell CD8 function and activity. Moreover, the expression of LIN7A gene showed a significant positive correlation (correlation coefficient >0.5) with the infiltration levels of Eosinophils and Monocytes (Supplementary Fig. 2C).

## 4 Discussion

### 4.1 Differential gene expression and pathway analysis reveals potential mechanisms in OA development

OA is a common degenerative joint disease characterized by cartilage degradation, inflammatory arthritis, and joint dysfunction. Bioinformatics methods have played an important role in unraveling the pathogenesis of OA and identifying potential therapeutic targets [20]. Bio-network analysis involves constructing and analyzing gene regulatory networks, protein-protein interaction networks, and signaling pathway networks to reveal key regulatory modules and interactive networks in OA [21,22]. By using databases such as GO and KEGG, it is possible to identify biological processes, pathways, and functional modules related to OA [23]. This helps further confirm key biological characteristics and molecular mechanisms involved in OA development. Bioinformatics analysis, utilizing DEGs, enhances our understanding of gene regulatory changes in OA and aids in uncovering associated signaling pathways and biological processes [[24], [25], [26]]. A gene expression profiling study has found significant alterations in gene expression related to chondrocyte differentiation and inflammatory response in OA cartilage tissue [27]. Several genes have been reported to be upregulated in OA in previous studies. For example, certain cytokines such as interleukin-1β (IL-1β), tumor necrosis factor-α (TNF-α), and interleukin-6 (IL-6) have been identified as key regulators in OA development and arthritis inflammation [28,29]. The high expression of these inflammatory mediators in OA joints leads to sustained activation of the inflammatory response, resulting in cartilage degradation and disease progression. Furthermore, the expression of some ECM-related genes (e.g., COL2A1, ACAN) has been found to be upregulated in OA [30], possibly reflecting the process of cartilage degradation and remodeling.

The exploration of OA's molecular mechanisms was conducted using bioinformatics methods, revealing the biological features of OA through DEGs and enrichment analysis. DEGs analysis revealed 1591 differentially expressed genes between normal samples and OA cartilage tissue samples, including 950 upregulated genes and 641 downregulated genes. PCA analysis, volcano plots, and heatmaps further displayed the pronounced differences in DEGs among the samples. GO enrichment analysis revealed that DEGs are mainly involved in biological processes such as ECM binding, immune receptor activity, and cytokine activity, affecting processes including humoral immune response, osteoblast differentiation, leukocyte migration, and reactive oxygen species metabolism. KEGG enrichment analysis showed that DEGs are primarily enriched in signaling pathways such as cytokine receptors, ECM-receptor interactions, and PI3K-Akt. Further GSEA analysis revealed that compared to the normal group, pathways such as AMPK, B-cell receptor, IL-17, and PPAR are inhibited in the OA group, while calcium signaling pathway, cell adhesion molecules, ECM-receptor interactions, TGF-β signaling pathway, and Wnt signaling pathway are significantly activated. In conclusion, our research results indicate a series of biological features related to ECM, immune response, cytokine activity, and multiple signaling pathways in OA.

Our analysis demonstrated that ANTXR1, KCNS3, SGCD exhibited significantly higher expression in female samples compared to male samples. As for the potential causes for differential gene expression between men and women in the context of OA, hormonal differences emerged as a pivotal factor [31]. The decline in estrogen levels, particularly postmenopausal, may contribute to an elevated risk of OA. Additionally, the presence of sex chromosomes (XX in females, XY in males) introduces differential gene dosage compensation, influencing gene regulation on the X and Y chromosomes [32]. In summary, our research not only delves into the intricate molecular mechanisms of OA but also establishes a connection between hormonal differences and sex-specific gene expression patterns. Further research is imperative to unveil the specific mechanisms underlying these differences and their implications for personalized approaches in managing OA.

These findings contribute to a better understanding of the molecular mechanisms underlying OA and provide potential targets and approaches for future development of OA therapies. Further research can be conducted to validate the functional and regulatory networks of DEGs and enrichment analysis results to further confirm these findings and explore their specific implications in the development and treatment of OA.

### 4.2 Identification of key genes and their diagnostic value in OA

WGCNA is a systems biology method used to construct gene co-expression networks and identify gene modules associated with specific diseases or biological processes. It utilizes gene expression data to calculate the correlation between genes and cluster highly correlated genes into modules, revealing gene regulatory networks and key regulatory genes [33]. Previous studies have employed WGCNA to analyze gene expression data, identify gene modules related to OA [33,34], and perform functional enrichment analysis to understand the biological mechanisms of OA. Machine learning algorithms and gene expression data have been integrated to identify potential diagnostic biomarkers with predictive value for OA [26,35]. In this study, RNA sequencing data from OA patients were analyzed using the WGCNA method to identify OA-associated genes. By intersecting DEGs with genes in key modules, 187 overlapping genes were selected as potential candidate genes associated with OA. Through LASSO logistic regression analysis, the study identified seven most predictive feature genes: LIN7A, KIF25, MYOM3, ANTXR1, KCNS3, SGCD, and C20orf3. Among them, KCNS3 showed the highest correlation with OA. Further analysis revealed the differential expression of these feature genes among different clinical groups. A column line plot model was constructed to successfully predict the association of these genes with OA and evaluate the diagnostic performance of the feature genes and the column line plot model. Validation confirmed the high expression of ANTXR1, KCNS3, and SGCD in cartilage tissue, blood, and synovial tissue, further supporting their significance in OA. Additionally, analysis of immune cell infiltration demonstrated a negative correlation between feature genes ANTXR1, KCNS3, SGCD, and T cell CD8, while LIN7A showed a positive correlation with the infiltration levels of Eosinophils and Monocytes. ANTXR1 may participate in cell adhesion and signaling, potentially influencing the pathogenesis of OA in joint cells [36]. Cell adhesion is fundamental for the structural integrity and function of joint tissues, and disruptions in cell adhesion may lead to changes in chondrocyte behavior and distribution within the cartilage [37]. KCNS3 encodes a potassium channel protein, which is crucial for maintaining the resting membrane potential of cells, influencing their excitability [38]. Changes in the electrophysiological properties of joint cells, such as chondrocytes, may impact cellular functions related to OA. Nevertheless, the causal relationship between these genes and osteoarthritis remains ambiguous. Further research is required to elucidate and clarify the existing uncertainties.

This study utilized the WGCNA method to construct a co-expression module network and identified the orange-red module as a key module associated with OA. Through differential gene expression screening and LASSO logistic regression analysis, seven most predictive feature genes were determined, revealing their association with immune cell infiltration. These findings provide valuable information for a deeper understanding of the pathogenesis of OA, identification of new therapeutic targets, and potential biomarkers for OA diagnosis.

## 5 Conclusion

Bioinformatics methods have played a crucial role in elucidating the pathogenic mechanisms of OA and identifying potential therapeutic targets. Through intersecting analysis, we identified 187 candidate genes associated with OA and used LASSO logistic regression to determine seven highly predictive feature genes. These feature genes include LIN7A, KIF25, MYOM3, ANTXR1, KCNS3, SGCD, and C20orf3, with KCNS3 showing the strongest correlation with OA. Further validation revealed differential expression of these feature genes across different clinical groups and evaluated their diagnostic value for OA. Validation results also demonstrated the high expression of ANTXR1, KCNS3, and SGCD in OA-related tissues and their correlation with immune cell infiltration. These research findings provide valuable insights into the molecular mechanisms underlying OA, potential therapeutic targets, and the development of potential diagnostic biomarkers for OA.

## Additional information

No additional information is available for this paper.

## Interest of conflict

The authors have no funding and conflicts of interest to disclose.

## Consent for publications

The authors read and proved the final manuscript for publication.

## Funding

None.

## Data availability statement

Data under study are available on request from the corresponding author on reasonable request. Datasets used in the study (GSE51588, GSE114007, GSE12021_GPL96, GSE55457, GSE82107, GSE63359) can be downloaded without restriction from the public GEO database.

## CRediT authorship contribution statement

**KunPeng Wang:** Conceptualization, Data curation, Writing – original draft. **Ye Li:** Data curation, Formal analysis, Writing – original draft. **JinXiu Lin:** Data curation, Formal analysis, Software, Writing – review & editing.

## Declaration of competing interest

The authors declare that they have no known competing financial interests or personal relationships that could have appeared to influence the work reported in this paper.
