# Supplementary material for: Identification of diagnostic biomarkers for osteoarthritis through bioinformatics and machine learning
Source: Heliyon. 2024 Mar 7;10(6):e27506. doi: 10.1016/j.heliyon.2024.e27506 (PMC10944228; doi:10.1016/j.heliyon.2024.e27506)
Supplement: Multimedia component 1 [file mmc1.docx]

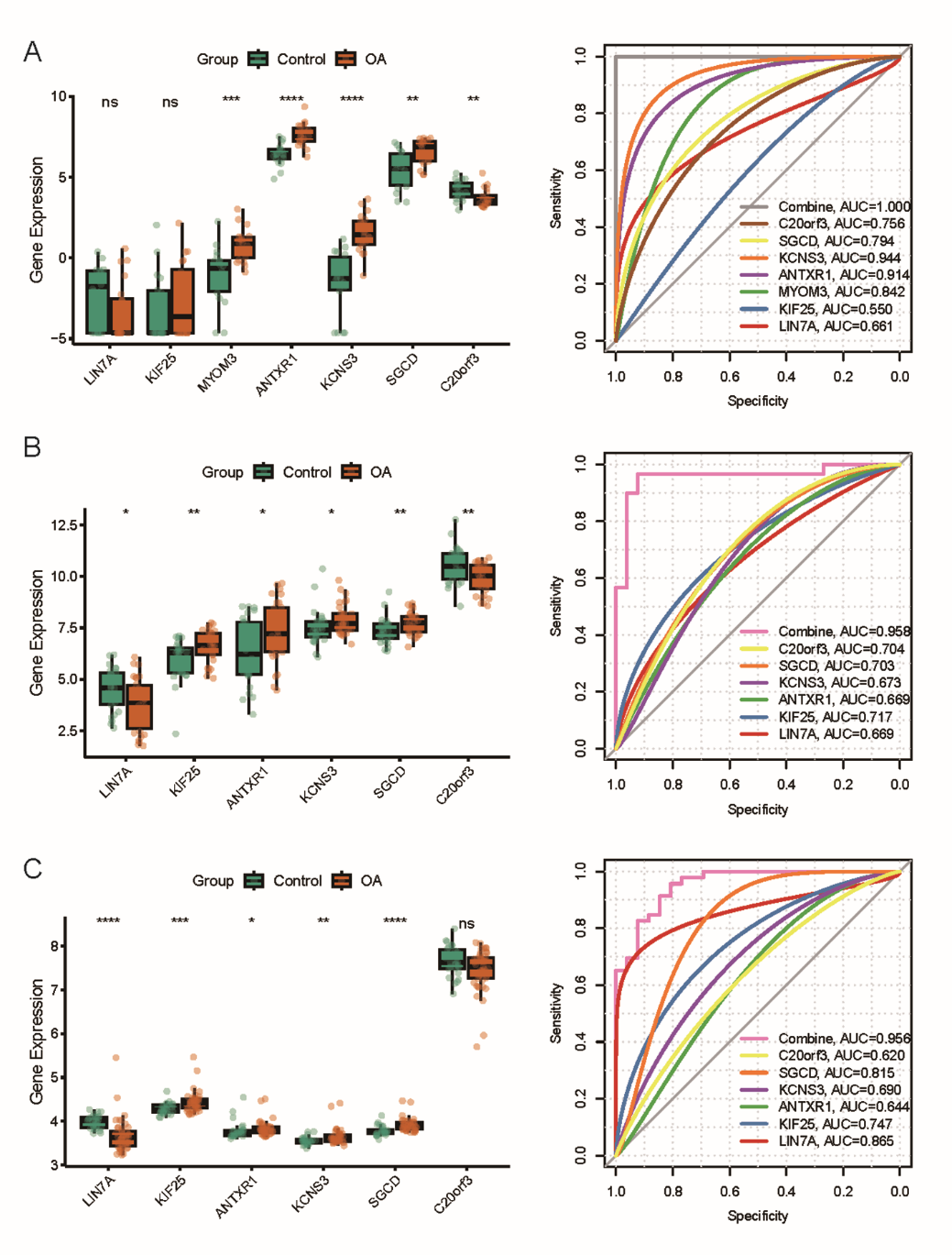


**Supplementary Fig. 1.** Validation of OA feature genes; (A) Validation results using the GSE114007


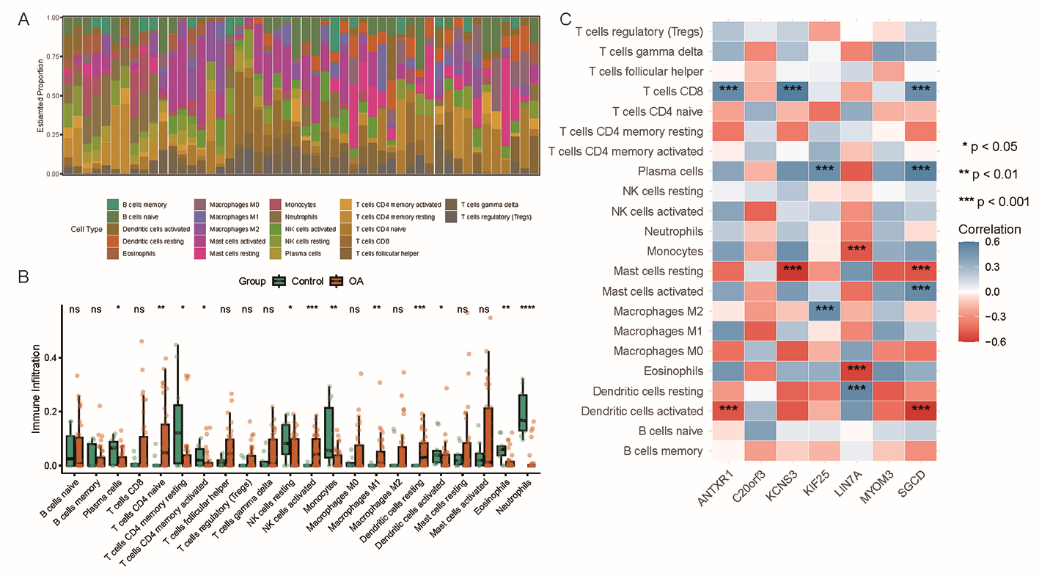


**Supplementary Fig. 2.** Association between OA feature genes and immune cell infiltration; (A) Proportions of 22 immune cell infiltrations in each sample from the GSE51588 dataset; (B) Differences in immune cell infiltration between normal control and OA articular cartilage samples; (C) Correlation between the expression of feature genes and immune cell infiltration.
